# Analyzing spatial clustering and the spatiotemporal nature and trends of HIV/AIDS prevalence using GIS: the case of Malawi, 1994-2010

**DOI:** 10.1186/1471-2334-14-285

**Published:** 2014-05-23

**Authors:** Leo C Zulu, Ezekiel Kalipeni, Eliza Johannes

**Affiliations:** 1 Department of Geography, Michigan State University, Geography Building, Auditorium Road, East Lansing, MI 48824, USA; 2 Department of Geography, 216 Ho Science Center, Colgate University, 13 Oak Drive, Hamilton, NY 13346, USA; 3 Institute for Defense Analyses, 4850 Mark Center Drive, Alexandria, VA 22311-1882, USA

**Keywords:** HIV/AIDS, GIS, Spatiotemporal analysis, Clustering, Hotspots, Coldspots, Drivers, Malawi, Africa

## Abstract

**Background:**

Although local spatiotemporal analysis can improve understanding of geographic variation of the HIV epidemic, its drivers, and the search for targeted interventions, it is limited in sub-Saharan Africa. Despite recent declines, Malawi’s estimated 10.0% HIV prevalence (2011) remained among the highest globally. Using data on pregnant women in Malawi, this study 1) examines spatiotemporal trends in HIV prevalence 1994-2010, and 2) for 2010, identifies and maps the spatial variation/clustering of factors associated with HIV prevalence at district level.

**Methods:**

Inverse distance weighting was used within ArcGIS Geographic Information Systems (GIS) software to generate continuous surfaces of HIV prevalence from point data (1994, 1996, 1999, 2001, 2003, 2005, 2007, and 2010) obtained from surveillance antenatal clinics. From the surfaces prevalence estimates were extracted at district level and the results mapped nationally. Spatial dependency (autocorrelation) and clustering of HIV prevalence were also analyzed. Correlation and multiple regression analyses were used to identify factors associated with HIV prevalence for 2010 and their spatial variation/clustering mapped and compared to HIV clustering.

**Results:**

Analysis revealed wide spatial variation in HIV prevalence at regional, urban/rural, district and sub-district levels. However, prevalence was spatially leveling out within and across ‘sub-epidemics’ while declining significantly after 1999. Prevalence exhibited statistically significant spatial dependence nationally following initial (1995-1999) localized, patchy low/high patterns as the epidemic spread rapidly. Locally, HIV “hotspots” clustered among eleven southern districts/cities while a “coldspot” captured configurations of six central region districts. Preliminary multiple regression of 2010 HIV prevalence produced a model with four significant explanatory factors (adjusted R^2^ = 0.688): mean distance to main roads, mean travel time to nearest transport, percentage that had taken an HIV test ever, and percentage attaining a senior primary education. Spatial clustering linked some factors to particular subsets of high HIV-prevalence districts.

**Conclusions:**

Spatial analysis enhanced understanding of local spatiotemporal variation in HIV prevalence, possible underlying factors, and potential for differentiated spatial targeting of interventions. Findings suggest that intervention strategies should also emphasize improved access to health/HIV services, basic education, and syphilis management, particularly in rural hotspot districts, as further research is done on drivers at finer scale.

## Background

### Introduction

**Figure 1:**
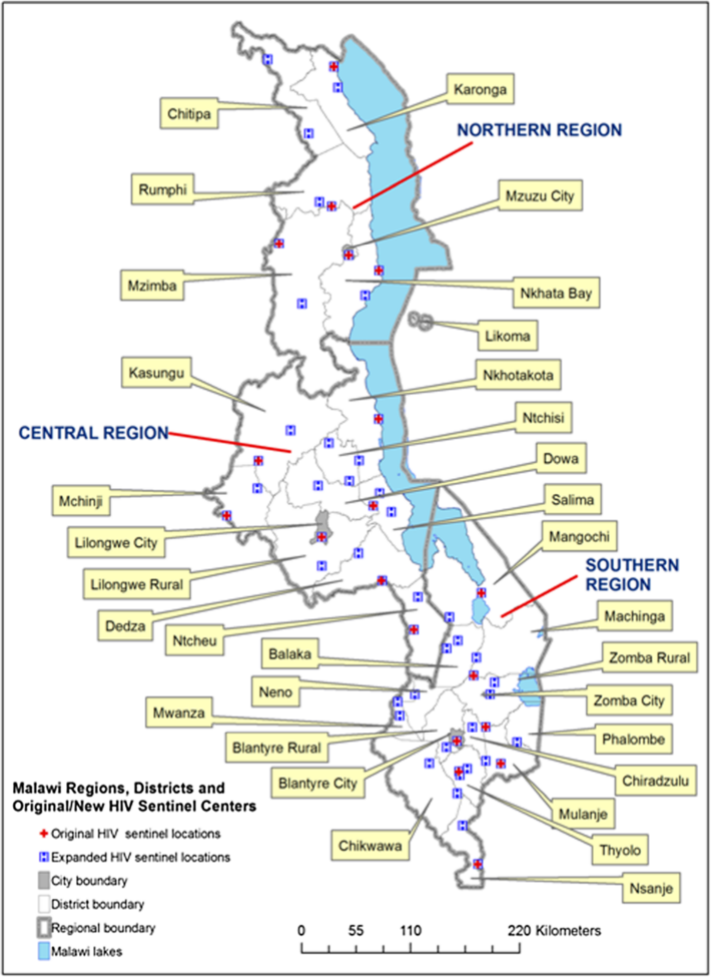
**Malawi’s administrative boundaries and location of sentinel antenatal clinic.** The figure shows the original 19, expanded 54 (in 2007), and overlapping ANC network for HIV surveillance. It also shows Malawi’s three regions, 28 districts, and four major cities, but Likoma (Island) was left out of the district regression analysis.

Understanding the nature and causes of spatial variation in HIV prevalence is essential to understanding and addressing the epidemic. Geospatial analytical methods, including geographic information systems (GIS), are an essential tool for achieving this. Yet even with the significant increase in the use of such geospatial tools in understanding public health problems in planning and implementing interventions and assessing their outcomes, geographically explicit studies of HIV/AIDS in sub-Saharan Africa are still very limited [1-3]. Existing studies are limited predominantly to coarse continental or cross-country analyses (e.g., [2,4-6]). Reasons for limited GIS use include scarcity of reliable spatially coded data. While a detailed review of the GIS/public health literature is beyond the scope of this article (but see [7-10]), Nykiforuk and Flaman [11] identify four categories of GIS use from a review of 621cases published between 1990 and 2007, namely: disease surveillance, risk analysis, access to health services and planning, and profiling community-health service utilization. Our study fits the surveillance and, partly, risk-analysis categories. Malawi provides a fitting setting because it is one of the six low-income countries with the highest HIV-prevalence rates globally [12], and requires more effective interventions even with recent declines in prevalence. Understanding spatio-temporal patterns of the HIV epidemic(s) in Malawi is limited to broad regional characterizations or urban/rural differences (e.g., [13-15]), and spatially differentiated knowledge of the underlying drivers is limited [3,8].This study uses primarily HIV prevalence data for pregnant women attending antenatal clinics (ANCs) in Malawi and spatial statistical tools to: 1) examine spatiotemporal trends and clustering of HIV prevalence in Malawi from 1994 to 2010, and 2) identify for the year 2010 variables associated with HIV prevalence and map their spatial clustering and variation relative to HIV “hotspots” and “coldspots”. We use women’s HIV data from 19 ANCs to address objective 1 for the selected years 1994, 1996, 1999, 2001, 2003, 2005, 2007, and 2010; and data from 54 ANCs to address objective 2 for 2010 (See Figure 1). Most notably, this study maps the spatial distribution and clustering of the identified factors in order to begin matching configurations of explanatory variables with particular clusters of HIV hotspots at district level for potential spatial targeting of HIV interventions in Malawi.

The 2012 global AIDS report placed Malawi among 25 countries with declines of 50% or more in new cases of adult (age 15-49) HIV infection globally. Malawi is also among countries reporting positive behavioral change or covering 60-79% of eligible people in antiretroviral therapy (2001- 2011), and is among 32 countries with a 25-49% decline in HIV deaths (2005-2011) [12]. Yet at 10.0% in 2011 [12,16], HIV prevalence remains a formidable challenge 26 years after the first diagnosis in1985. Among sex workers, prevalence was up to 70%, while 923,058 of Malawi’s 14 million people were living with HIV/AIDS in 2010 [13]. Nearly 600,000 children were orphaned due to HIV/AIDS [17], and there are glaring gender and urban/rural disparities. HIV prevalence was 2.2 times higher among female than male youth aged 15-24, and 2.8 times higher among urban women than their rural counterparts (22.7% versus 10.5%) in 2010 [14]. Further, decline in HIV *prevalence* in the general population has been modest – from 11.8% in 2004 to 10.6% in 2010. As with other epidemics, understanding such spatial variation in HIV/AIDS prevalence and its drivers within a social, spatial and temporal context is crucial for spatial targeting of interventions and resources [1,7,18].

This article is divided into five parts. First, we briefly review the literature on the use of GIS and spatial analysis in analyzing HIV prevalence, and on commonly found drivers of HIV risk and prevalence. A presentation of broader spatiotemporal patterns of HIV prevalence at national and regional scale, including the significant urban/rural divide, follows. Third, the overall spatial structure of the epidemic is examined based on spatial statistical analysis of the presence, nature, temporal trends and implications of spatial dependency in prevalence rates. This knowledge is used to decompose spatial patterns of HIV prevalence first into continuous surfaces using spatial interpolation techniques in order to show continuous spatiotemporal variation nationally, and second at district level after spatial aggregation (averages). The fourth section presents analysis of local clustering patterns, or “hotspots” and “coldspots,” of HIV prevalence at district level.^a^ The fifth part presents models of indicative drivers of HIV prevalence identified from multiple regression analysis and mapping of the spatial variation and clustering of the drivers. Study findings and implications are then discussed, and limitations presented before offering a concluding section. The methodology used is presented at the end.

### Use of GIS and geospatial analytical methods in understanding HIV prevalence

Among the limited but growing uses of GIS in HIV analyses in Africa, very few studies have analyzed spatiotemporal variation in HIV prevalence, including clustering patterns, at meso (e.g., district) or lower scales, where better understanding can enhance the effectiveness of interventions [19].^b^ Occasional studies during early parts of the epidemic demonstrated the value of geographic analysis in understanding HIV prevalence, risk, and spread; including in mapping the distribution of at-risk populations of commercial sex workers and uncircumcised males [20,21]. However, most of the few recent GIS-based analyses of HIV/AIDS in Africa have been mainly at coarse continental scale with limited national policy relevance (e.g., [5,6]), or at fine local scales requiring heavy data collection/use but with a limited spatial scope, e.g., assessment of local HIV variability/clustering and risk in a rural sub-district in northern KwaZulu-Natal Province, South Africa [1,22].^c^ Very few exceptions have analyzed country-wide spatial variation and clustering of HIV prevalence, and their drivers. Messina et al. [3] conducted such a study involving sex differentiated spatial variation in HIV prevalence using 2007 demographic and health survey (DHS) data, GIS, and regression analysis of HIV drivers at “community” (neighborhood or village-clusters) scale in the Democratic Republic of the Congo (DRC). Moise and Kalipeni [23] recently used GIS and HIV sentinel data for pregnant women attending ANCs in Zambia to analyze spatiotemporal patterns of HIV prevalence from 1994 to 2004. They also used spatial statistical regression modeling to identify possible drivers of HIV prevalence for 2004 at the district scale, where health-related data are reported and planning and decision-making done. Despite limitations involving sample representativeness and paucity of data compared to population-based (e.g. DHS) HIV data, ANC data are more readily available, allow longitudinal trend analysis, and can be re-scaled to district or other scales using spatial interpolation techniques [2,6]. Our study uniquely goes beyond HIV “hotspot” analysis to map the spatial variation of likely drivers allowing identification of configurations of explanatory variables for particular clusters of HIV hotspot districts for more effective intervention targeting.

### Common drivers of HIV/AIDS prevalence

HIV epidemics are diverse and complex to deal with because their drivers are numerous, diverse and vary over space and time. Given that most HIV transmission in sub-Saharan Africa is through heterosexual intercourse, the major proximate driver is known - having unprotected sex with an infected person, and the higher the number of cumulative and concurrent sexual partners, the higher the risk of transmission [12,14,24]. Yet recent research shows that there are underlying HIV drivers that go deeper than biomedical or narrow epidemiological susceptibility factors. These include diverse socio-economic, demographic, cultural, historical, and geographic factors and their configurations that affect the vulnerability of particular groups of men and women who engage in such risky sexual behavior to HIV infection [25-29].

For Malawi, reported underlying HIV drivers have included high levels of poverty (and wealth), low literacy, high rates of unprotected casual and transactional sex, low male and female condom use, cultural (e.g., widow cleansing and/or ‘hyena customs’) and religious factors, gender inequity and low social and economic status of women, high-risk livelihoods, high migration/mobility levels, high incidences of sexually transmitted diseases and tuberculosis, and geographic factors mainly involving poor access to health services or increased exposure to risks. For instance, several curable sexually transmitted diseases can increase the risk of HIV transmission 2-20 times per sexual contact, and prevalence is higher among those with poor access to prompt treatment [15]. Poverty has forced some women into commercial sex or other risky sexual behavior in order to survive, increasing their risk of contracting/spreading HIV/AIDS [30,31]. Therefore, conditions of high unemployment, and low and insecure wages that lead to such behavior may help explain high HIV rates among the urban poor. Tough economic conditions have also driven international male labor migration, including into mining hubs in South Africa where men are separated from their spouses for extended time periods and vulnerable to casual sex and HIV infection, and spreading HIV in their areas on their return [32,33]. Geographically, high HIV infection has been associated with close proximity to major transportation networks which have provided transmission arteries for HIV within and across countries, and to urban and trading centers and transport networks linking them [3,6,34].

## Methods

### HIV prevalence data and temporal trends at national, regional and urban/rural scales

HIV/AIDS data came from mean HIV prevalence rates among pregnant women attending a longitudinally rich network of 19 ante-natal clinics (ANCs) where HIV surveillance has been conducted from 1994 to 2010. In 2007, the network was nearly tripled to 54 ANCs while maintaining the original 19, allowing continuity and spatiotemporal analysis (Figure 1). Data included the location (latitude and longitude coordinates) of the ANCs, allowing their mapping. HIV data collection frequency was annual from 1994-1999 and subsequently largely biennial. Although limited demographic information is also collected, only HIV prevalence was available to the authors for all the years, sourced from U.S. based Centra Technology Inc. (1994-2003) and HIV/syphilis surveillance reports produced by Malawi’s National AIDS Commission, NAC [13,35,36]. Malawi uses standard sampling, HIV testing, and data analysis methods and models recommended by the Joint United Nations Program on HIV/AIDS (UNAIDS) and the World Health Organization (WHO) (e.g., [37,38]).^d^ Despite known limitations of such sentinel based HIV prevalence estimates [18,39], they remain the major source of HIV data in Malawi and other African countries, and the only longitudinal record for analyzing the spatiotemporal *trends* targeted in this study without projecting to the general population or generating predictions.^e^

### Spatial dependence in HIV prevalence, spatial interpolation, and spatiotemporal trends

First, we plotted HIV prevalence rates for pregnant women 15 to 49 years old [13,17] from 1995 to 2010) to provide a broad, multi-scalar, spatiotemporal perspective of the HIV epidemics at national, regional, urban and rural scales. Then GIS tools were used to 1) empirically test for spatial dependency in HIV prevalence nationally, 2) produce a continuous surface of HIV prevalence at 1 × 1 km spatial resolution for visualization and generation of prevalence estimates at district level for cluster/hotspot and regression analysis. GIS analysis was conducted with AcrGIS desktop 10.0 (Redlands, CA: Environmental Systems Research Institute, Inc., 1999).

The presence and nature of HIV spatial autocorrelation (or dependency) was assessed empirically for each of the available 17 data years spanning 1994 to 2010 for the original 19 ANCs, using the global Moran’s I statistic [40]. The presence of spatial autocorrelation can suggest HIV clustering, sometimes indicative of hierarchical expansionary spread in urban areas and across districts [41]. Moran’s I is based on Waldo Tobler’s first law of geography: “everything is related to everything else, but close things are more related than distant things” [42]. If this law applies, HIV prevalence rates should be similar among neighboring districts than among non-neighbors. Moran’s I tests the null hypothesis that measured values at one location are independent of values at other locations (i.e., HIV prevalence is randomly dispersed). Its value varies from -1 to 1. Positive values indicate presence of spatial autocorrelation, zero means total spatial randomness, and negative values indicate dissimilar values clustered next to one another. A statistically significant Moran’s I (p < 0.05) leads to rejection of the null hypothesis and indicates the presence of spatial autocorrelation. Global Moran’s I is computed as follow:

$$I=\frac{N\sum_{i}\sum_{j}w_{\mathrm{ij}}\left(X_{i}‒\overset{¯}{X}\right)\left(X_{j}‒\overset{¯}{X}\right)}{\left(\sum_{i}\sum_{j}w_{\mathrm{ij}}\right)\sum_{i}{\left(X_{i}‒\overset{¯}{X}\right)}^{2}}$$

where N is the number of spatial units (sentinel ANCs), X_i_ is the measured value for feature I (up to N), X_j_ is the measured value for a neighboring point j (up to N-1), and W_ij_ represents a weight measure of the influence of neighboring feature j on measured value at I derived from the row-standardized spatial weight matrix.

In order to produce smooth surfaces of HIV prevalence for visualization and data generation at district level, the Inverse Distance Weighted (IDW) spatial interpolation method was used for the selected years (1994, 1996, 1999, 2001, 2003, 2005, 2007 and 2010). These years were chosen for trend continuity while including years of positively autocorrelated HIV prevalence (partly justifying use of IDW) and early years (1996, 1999, 2001) of non-significant and/or negative autocorrelation which nevertheless illuminate spatiotemporal patterns. Spatial interpolation methods apply mathematical models to measured point values of a continuous variable at known locations to predict values at locations that do not have values, thereby creating a continuous surface [43,44]. In predicting values, interpolation methods generally use distance-based weights that assign more influence to measured values nearest an unmeasured location than to measured values located farther away. *Deterministic* interpolators, including IDW, use weights based only on distance between measured and unmeasured points while *geostatistical* (or stochastic, e.g., kriging) use sophisticated weights combining distance with probabilistic statistical models of the spatial variation among measured points. IDW produced stable and reasonably reliable predictions for cross-year comparisons with the small sample size (from 19 ANCs). It has been used reliably with small-medium samples in HIV studies [2,23], at times preferred over (potentially superior) kriging whose performance often suffers more with small samples because of probability distribution requirements [44,45]. With IDW, we used a variable setting of 6-10 points to predict values at each unknown location based on iterative testing to minimize mean error and root mean square error (RMSE). We then used GIS tools to extract HIV estimates for the 31 ‘districts’ (27 of Malawi’s 28 districts and four main cities of Blantyre, Lilongwe, Zomba and Mzuzu, Figure 1) by averaging prevalence values in constituent 1 × 1 km spatial cells.

### Local spatiotemporal variation in HIV prevalence and cluster/‘hotspot’ analysis

Two local measures of spatial association were used within ArcGIS 10.0 to indicate “where the clusters or outliers are located” and “what type of spatial correlation is most important” [46]. Anselin Local Moran’s I [46] allowed us to detect core clusters/outliers of districts with extreme HIV prevalence values unexplained by random variation, and to classify them into hotspots (high values next to high, HH), “coldspots” (low values next to low, LL) and spatial outliers (high amongst low, HL or vice versa, LH). Local Moran’s I tests the same null hypothesis of absence of spatial dependence (for polygon features) when its expected value is -1/(N - 1). It has been used in studies to identify HIV hotspots [15,18,23]. Further, the local Getis-Ord statistic, Gi* was used to provide additional information indicating the intensity and stability of core hotspot/coldspot clusters [47,48]. The statistical significance of a Z-score assigned to each district identified the presence and intensity of local clusters of hotspots and coldspots of HIV prevalence within a radius of 80 km, relative to the hypothesis of spatial randomness. This fixed distance, identified iteratively as maximizing autocorrelation (global Moran’s I) and maintaining stability across years [44], defined the neighborhood search for a particular district, including for analysis with Anselin’s Local Moran’s I. The Getis-OrdGi* index is calculated as:

(1) $$G_{i}^{*}=\frac{\overset{n}{\underset{j=1}{\sum }}w_{i,j}x_{j}‒\overset{¯}{X}\overset{n}{\underset{j=1}{\sum }}w_{i,j}}{{}_{S}\sqrt{\frac{n\overset{n}{\underset{j=1}{\sum }}w_{i,j}^{2}‒{\left(\overset{n}{\underset{j=1}{\sum }}w_{i,j}\right)}^{2}}{n‒1}}}$$

Where *x*_*j*_ is HIV prevalence for district *j*, *w*_*i,j*_ is the spatial weight between districts *i* and *j*, *n* is the total number of districts (31), and

(2) $$\overset{¯}{X}=\frac{\overset{n}{\underset{j=1}{\sum }}x_{j}}{n}$$

(3) $$S=\sqrt{\frac{\overset{n}{\underset{j=1}{\sum }}x_{j}^{2}}{n}‒{\left(\overset{¯}{X}\right)}^{2}}$$

### Regression analysis and indicative drivers of HIV prevalence for 2010

In order to identify and illustrate potential linking of indicative drivers of observed spatial variation of HIV prevalence to particular hotspot/coldspot clusters of districts, we conducted multiple regression analysis of HIV prevalence for 2010 only, and then mapped the spatial distribution and clustering of selected factors. HIV prevalence per district among pregnant women attending ANCs, the dependent variable, was estimated using GIS as explained earlier for cluster analysis, but using the latest sentinel HIV data (2010) from the 54 ANCs (instead of 19). The year 2010 also matched dates of available explanatory factors closely.

Independent variables were chosen based on the literature (background section) and availability at district level. Main sources of independent variables were national surveys conducted by the Malawi National statistical Office (NSO) and GIS-generated data. Surveys included the 1998 census, 2011 Welfare Monitoring Survey (WMS) and 2010/2011 Integrated Health Survey, IHS3 [49-51]. Variables included socio-demographic (e.g., education, poverty/wealth/consumption, population density and mobility, employment, often by age/sex), HIV awareness and behavior (value and use of condoms, self-reported HIV testing in 2010 or ever, and gap between awareness and behavior on HIV testing). Syphilis prevalence for 2010 was the only socio-biological variable used that was available to us [13]. Geographic variables were also used to address underlying factors related to access to HIV related amenities/services (distance/time to roads, public transport, and health facilities), mobility and exposure to higher risks (proximity to cities), and elevation, sourced from surveys or generated using GIS processing (Table 1). The starting pool of independent variables was 37. They are not necessarily the most important in explaining variation in HIV prevalence, but the subsequent multi-stage statistical screening process in correlation analysis and later stepwise regression analysis narrowed them down to some significant factors that adequately reflect observed spatial variation among pregnant women in the 31 districts of Malawi. All the data used in this study are publicly available, aggregated secondary data (see Table 1) which do not have any personal information or identifying information that can be linked to particular individuals or communities. Consequently, there were no significant ethical concerns, or approval (or permission) needed to use the data. All sources, however, have been acknowledged.

**Table 1:** Summary of selected variables significantly correlated with 2010 HIV prevalence

**Variable name**	**Variable type, description**	**Mean**	**Min.**	**Max.**	**SD**	**Pearson r**	**Variable source**
**Dependent variable**							
HIV_Y10	Estimated HIV prevalence, 2010 (%)	11.65	6.00	22.40	4.32	1.000	2010 HIV/syphilis report, NAC 2011
**Socio-demographic**							
POPDEN08^a^	Persons per km^2^, 2008	433.94	36.00	3006.00	797.20	0.474***	1998 census, NSO 2008
ED_S_PRIM^a^	Attended senior primary sch. (%)	29.068	19.70	45.10	6.180	-0.445***	WMS 2011, NSO 2012
MIGRGROSS^a^	Gross migration (in and out migration), 2008 (%)	35.839	19.00	102.00	18.817	0.376**	1998 census, NSO 2008
UNEMP_FEM^a^	Unemployed female, 2011 (%)	22.494	1.30	73.50	18.197	0.415*	WMS 2011, NSO 2012a
POPP25_49	Population, age 25_49, 2008 (%)	44.958	41.50	56.00	3.949	0.309**	1998 census, NSO 2008
URB_DISTR^a^	District share of total urban population, 2008 (%)	3.213	0.00	33.7.00	8.160	0.382**	1998 census, NSO 2008
CONSUME^a^	Per capita consumption, 2011 (MK)	54076	26645	152907	28017	0.46**	IHS3, NSO 2012b
UNEMP_MALE	Unemployed male, 2011 (%)	19.77	2.20	63.20	16.133	0.320	WMS 2011, NSO 2012a
**HIV awareness and behavior**							
TEST_EVER2^a^	Percentage that had taken an HIV test ever	66.542	51.50	84.50	7.833	510***	WMS 2011, NSO 2012a
HTEST_POSS2^a^	Percentage who know a confidential HIV test is possible	84.439	63.50	97.10	9.684	0.323*	WMS 2011, NSO 2012a
**Socio-biological**							
SYPHILIS^a^	Proportion of women positive for syphilis (%, 2010)	0.285	0.03	1.32	0.268	0.462***	2010 HIV, syphilis report, NAC 2011
**Geographic (proximity, access, exposure)**							
DIST_HF	Mean dist. to HF	18.915	4.00	38.60	8.790	-0.567***	GIS derived, 2001 HF data, MoH
Dist_HF30_44^a^	Mean dist. to HF, age 30_44	16.161	5.90	34.30	6.898	0.365**	WMS 2011, NSO 2012a
DIST_RD^a^	Mean dist. to main roads (km)	6.224	1.00	16.40	3.489	-0.605***	GIS based, roads layer from Malawi Surveys Dept.
DISTCITY^a^	Mean dist. to major city (km)	53.761	0.00	164.83	40.323	-0.540***	GIS based, cities map, NSO
T_RD30_44	Mean time to all-weather road, age 30_44 (min)	8.961	1.10	28.60	5.135	0.370**	WMS 2011, NSO 2012a
T_TRANS30_44^a^	Mean time to transport, age 30_44 (min)	12.329	4.40	25.20	4.882	0.396**	WMS 2011, NSO 2012a
DIST_HF45_59^a^	Mean dist. to HF, age 45_59	13.542	5.30	33.3	4.307	0.302*	WMS 2011, NSO 2012a

Variables were screened from an initial list of 37. Superscript ^a^indicates one of 13 variables entered in to the regression model. The superscripts *, **, and *** indicate that the correlation is significant at the 0.05, 0.01 and 0.00 level (2-tailed, respectively. HF is health facility, IHS3 is the Third (2010/11) Integrated Health Survey, MK is the Malawi Kwacha (Malawi’s currency), MoH is Ministry of Health, NAC is the Malawi National AIDS Commission, NSO is the Malawi National Statistical Office, and WMS is the Welfare Monitoring Survey.

Correlation analysis against HIV prevalence was used to screen the initial variable pool, yielding the 18 statistically significant (p ≤ 0.10) ones listed in Table 1 (the full list is available on request). We used further correlation analysis among the 18 to narrow the significant variables to 13 (variable names marked with the superscript ^**a**^ in Table 1) by removing highly correlated variables (generally r > 0.7, p ≤ 0.05). For instance, mean distance to health facilities was dropped because it was highly correlated (r = 0.784, p = 0.000) with distance to main roads but slightly less correlated with HIV prevalence. We entered the 13 independent variables into SPSS 20.0 (IBM SPSS Statistics for Windows, Version 20.0. Armonk, NY: IBM Corp.) for multiple regression using forward stepwise entry after standardizing them to Z values to stabilize variability and curb observed remnant multi-collinearity. Several collinearity diagnostics and partial significance statistics were used to further limitmulticollinearity problems and to pick a “best” model among the four produced.

Variables in the “best” model had to have a Variance Inflation Factor (VIF) below 2, Condition Index below 30, and tolerance values above 0.5 to signify non-significant collinearity. Additional diagnostics on the “best” model confirmed multi-collinearity and heteroskedasticity not to be significant problems. Cluster/hotspot analysis and mapping were performed on variables from the “best” model and ancillary correlation analysis used to explain observations.

The narrow focus of the second study objective on identifying and mapping spatial patterns of indicative explanatory factors of HIV prevalence, rather than producing predictive models, and their basis on the literature, should mitigate concerns over automated variable-selection methods [52]. For the same reasons, we maintained a simple ordinary least squares (OLS) model. Additional spatial diagnostics on the “best” OLS model using the GEODA spatial statistical software (GeoDa Center for Geospatial Analysis and Computation, Arizona State University) confirmed expected breach of the OLS independence assumption given the significant spatial dependence. Although the corrective spatial lag model [51,53] and significant of its autocorrelative coefficients and individual variable coefficients were generally an improvement on the OLS model, except a slight decline in the significance of one variable, it did not sufficiently change the essentials of the OLS model. Thus, we report only results of the OLS model for purposes of this study.

## Results

### Temporal and spatial trends in HIV prevalence at national and regional scales

**Figure 2:**
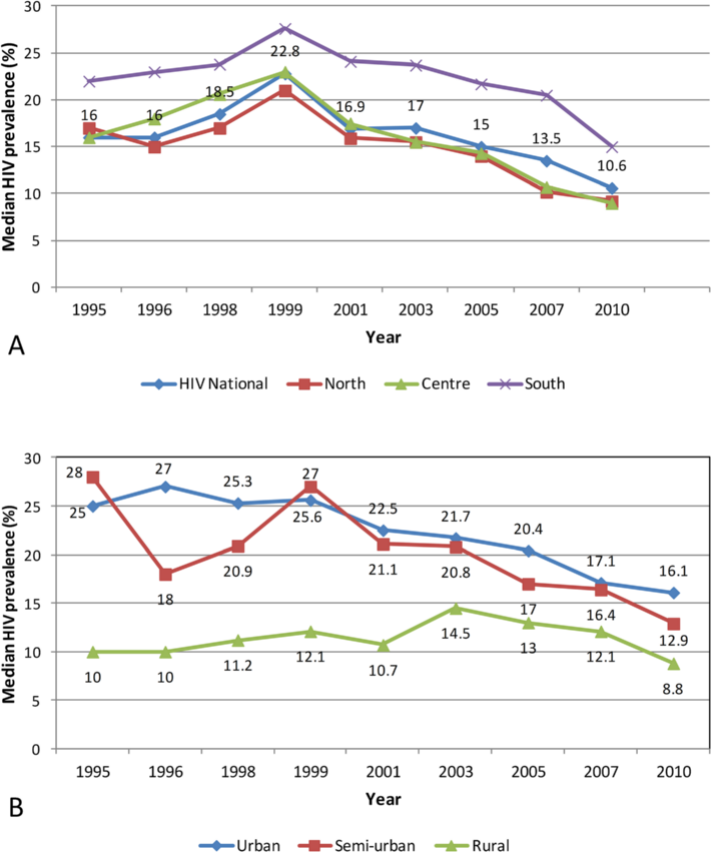
**National, regional, rural, and urban trends in HIV prevalence for pregnant women, 1995-2010. A** shows the national median prevalence rate (labels on chart, percent) relative to trends for the northern, central and southern regions. **B** shows temporal trends in HIV prevalence by residence type: urban, semi-urban and rural. Data sources: Government of Malawi 2012, NAC 2011.

In Malawi, two broad geographic trends emerge of HIV/AIDS prevalence among pregnant women attending ANCs: 1) a significant overall decline in prevalence since the peak of the epidemic in 1999, and 2) multiple geographically defined HIV ‘epidemics’ with diverse spatiotemporal trends. National median HIV prevalence increased from 16% in 1995 and peaked at 22.8% in 1999 before declining to 10.6% in 2010 – an average annual drop of 1.1% (Figure 2A). The Southern Region consistently had the highest HIV prevalence, of 7.0% higher than the national prevalence (1996 and 2007), but narrowing to a 4.4% gap by 2010 (Figure 2A). The Northern Region had the lowest before its trajectory essentially merged with the Central Region’s from 2003. An urban/rural divide contrasts a severe urban epidemic with a less intense and variable, and slower/lower peaking rural epidemic (Figure 2B). However, the intensity of the two epidemics has been converging from a 2.8-fold difference in HIV prevalence (28% urban versus 10% rural) in 1995 to 1.5-fold by 2010. The urban epidemic peaked earliest and highest (1996 at 27%), and declined slower (average 0.73% annually) than the national epidemic to 16.1% in 2010. The semi-urban epidemic varied considerably (1995 to 1999), and then settled below the urban trajectory. The rural epidemic was relatively stagnant between 10% and 15%.

### Spatial autocorrelation, GIS interpolation mapping and spatiotemporal trends

**Figure 3:**
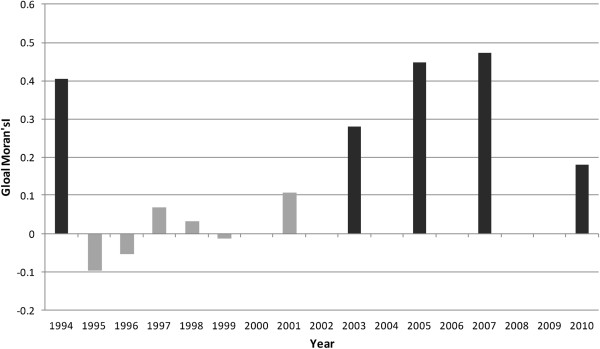
**Global Moran’s I and Spatial Dependence in HIV Prevalence, 1994-2010.** The black bars in Figure 3 show global Moran’s I values in years for which the statistic (and spatial autocorrelation) was statistically significant at p ≤ 0.01, the dark grey at p = 0.05 or p = 0.10), and the light grey bars for years with no statistical significance. The positive Moran’s I values indicate positive autocorrelation, i.e., HIV prevalence values at neighboring locations were similarly high or low, while negative values indicate negative autocorrelation with high prevalence values next to low ones.

**Figure 4:**
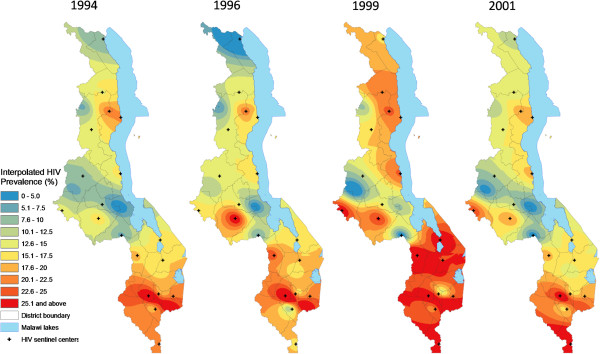
**Interpolated spatiotemporal trends of the HIV/AIDS Epidemic among pregnant women, 1994 – 2001.** Continuous images produced by interpolating (IDW method at 1 km spatial resolution) HIV prevalence (%) among pregnant women attending the original 19 HIV sentinel centers in 1994, 1996, 1999 and 2001.

**Figure 5:**
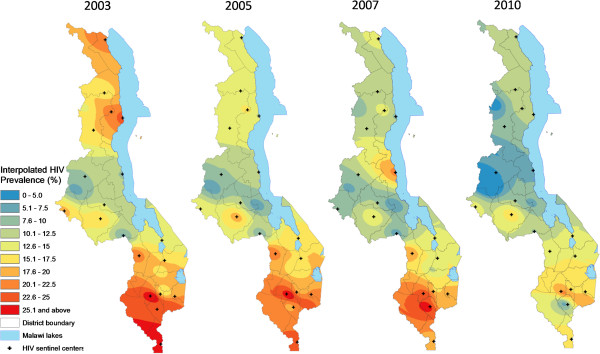
**Interpolated spatiotemporal trends of the HIV/AIDS Epidemic among pregnant women, 2003 – 2010.** Continuous images produced by interpolating (IDW method at 1 km spatial resolution) HIV prevalence (%) among pregnant women attending the original 19 HIV sentinel centers in 2003, 2005, 2007 and 2010.

**Figure 6:**
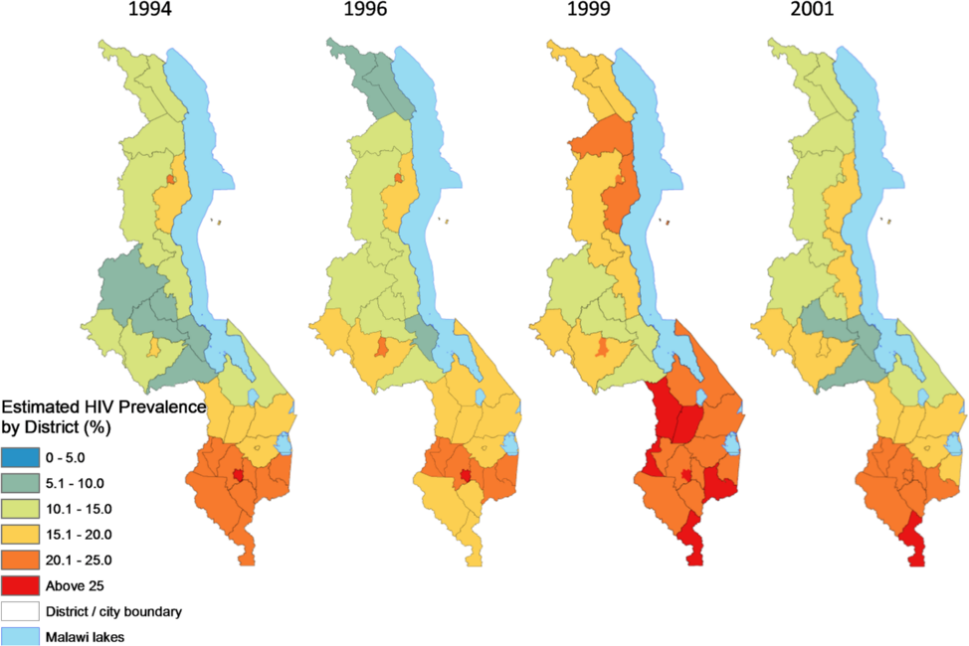
**Spatiotemporal trends in estimated average HIV prevalence among pregnant women by district, 1994-2001.** District/city estimates of HIV/AIDS prevalence were derived by averaging prevalence for all 1-km cells in the interpolated surfaces falling within each district and major city for 1994, 1996, 1999 and 2001. These rates are indicative and only for assessing spatial patterns and temporal change, rather than authoritative district estimates.

**Figure 7:**
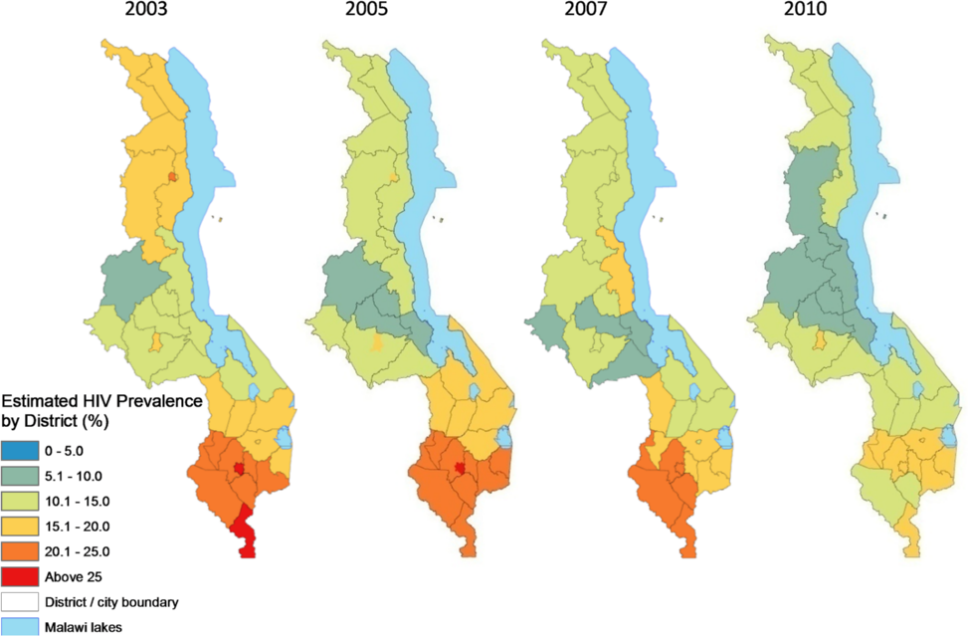
**Spatiotemporal trends in estimated average HIV prevalence among pregnant women by district, 2003-2010.** District/city estimates of HIV/AIDS prevalence were derived by averaging prevalence for all 1-km cells in the interpolated surfaces falling within each district and major city for 1994, 1996, 1999 and 200. These rates are indicative and only for assessing spatial patterns and temporal change. They are not authoritative district estimates.

Spatial analysis showed the presence of positive spatial autocorrelation (global Moran’s I > 0) in HIV prevalence among pregnant women for eight of the 11 available data years, five of them statistically significant (p < 0.01, see Figure 3) and confirming the presence of spatial structure. However, there was significant temporal variation in the spatial dependence of HIV prevalence, including early years (1995, 1996, 1999) years of negative autocorrelation followed by a general increase in the size and significance of Moran’s I (range -1 to 1) from -0.09 in 1999 to peak at 0.474 in 2007.Continuous surfaces of HIV prevalence (Figures 4 and 5) and district estimates extracted thereof (Figures 6 and 7) confirmed and spatially unpacked regional and urban/rural variation in HIV prevalence among pregnant women. In addition, Figures 4 and 5 capture general intensification of the HIV epidemic in prevalence and spatial extent from 1994 to 1999. HIV intensity attenuated gradually from above 25% in 1999 to generally below 12.5% nationally by 2010. Sub-epicenters emerged around Nkhata Bay district and Mzuzu city in the Northern Region and Lilongwe City and Mchinji district in the Central Regions, but had dissipated by 2003. District prevalence estimates (Figures 6 and 7) confirmed the persistently high rates in the south, general intensification from 1994 to 1999 and subsequent decline in prevalence and spatial extent of pockets of high/low prevalence.

**Figure 8:**
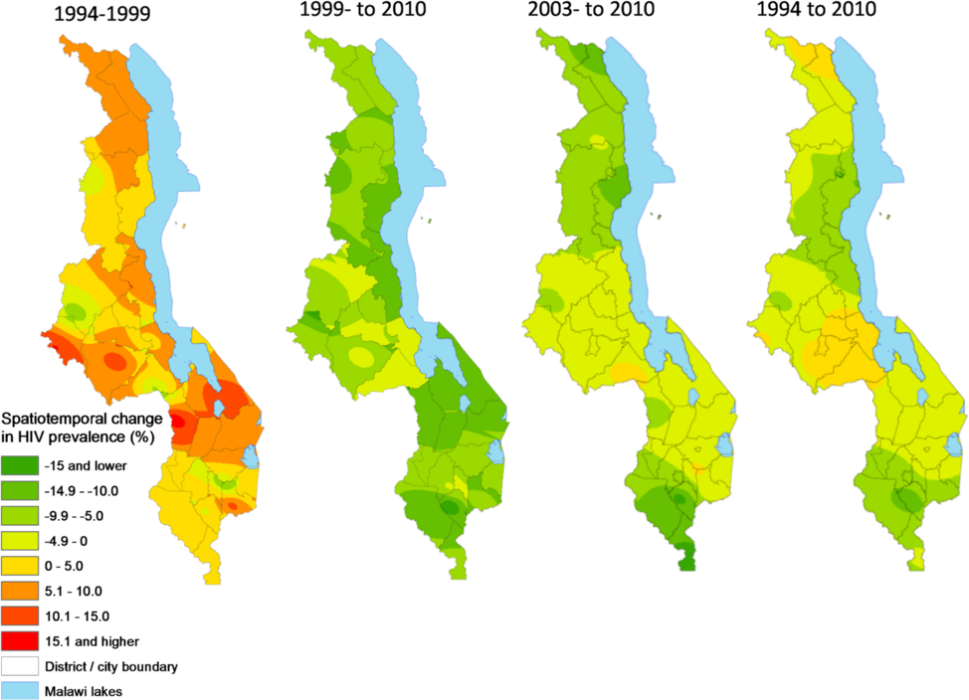
**Temporal change in continuous HIV prevalence among pregnant women for various periods between 1994 and 2010.** Negative values (shades of green) represent a decrease (%) in prevalence and positive values an increase, for the continuous surfaces for the time periods 1994-1999, 1999-2010, 2003-2010 and 1994-2010.

**Figure 9:**
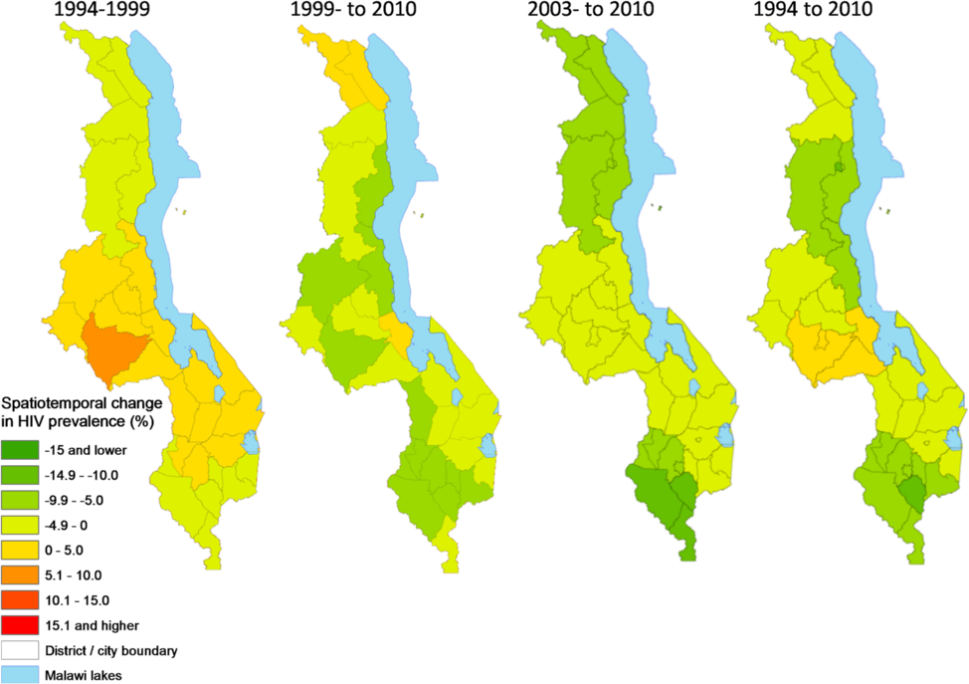
**Temporal change in district-level estimates of HIV prevalence among pregnant women for various periods between 1994 and 2010.** Negative values (shades of green) represent a decrease (%) in prevalence and positive values an increase, for the continuous surfaces for the time periods 1994-1999, 1999-2010, 2003-2010 and 1994-2010.

Change analysis based on continuous images (Figure 8) and district-level analysis (Figure 9) showed spatial variation across the four time periods analyzed dominated by the initial expansion (1994-1999) and subsequent decline in HIV prevalence. Most of the initial prevalence increases were in the Central Region and northern parts of the Southern Region, with pockets in the north. Overall, major decreases in HIV prevalence (darker green areas in Figures 8 and 9) had occurred mainly in the Southern Region, with some pockets in the Central and Northern regions. However, by the period 2003-2010, declines in HIV prevalence were clearly dominated by the Northern and Southern regions. Three Central Region districts (Lilongwe district/City, Dedza and Salima) had increased in HIV prevalence slightly by 2010 relative to 1994 while one central (Salima) and two northern (Chitipa and Karonga) districts had gained after 1999, peak of the epidemic.

### Local spatial variation and hotspot analyses of HIV prevalence

**Figure 10:**
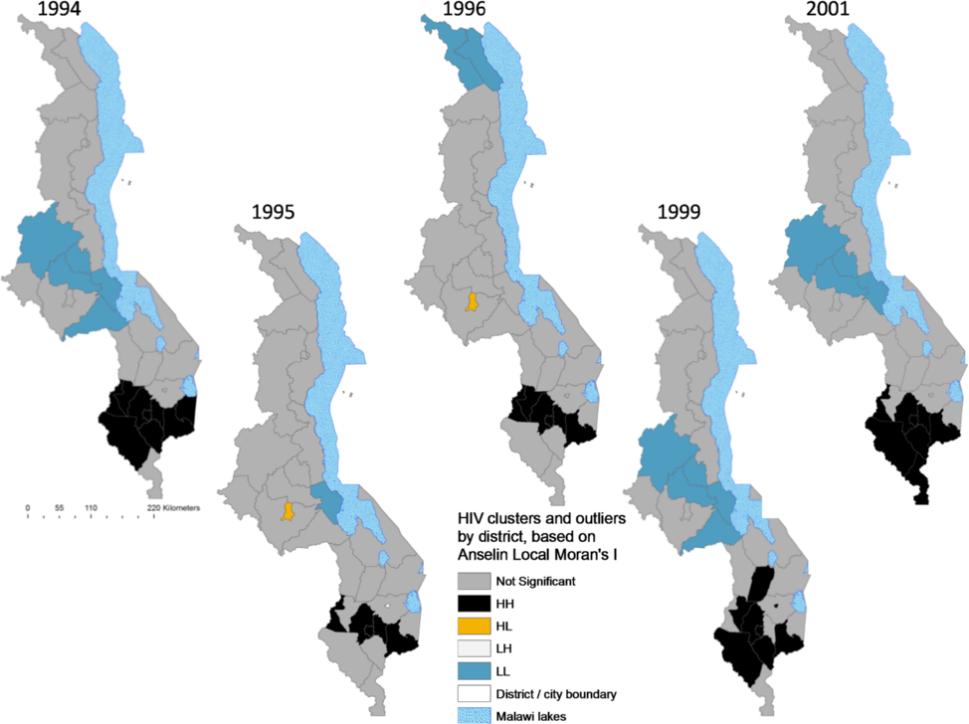
**Spatiotemporal patterns of HIV hotspots and outliers by district, 1994-2001.** Estimates of district HIV prevalence were based on the original 19 ANCs to allow longitudinal continuity from 1994 to 2010. Figure 10 shows the years 1994, 1995, 1996, 1999 and 2001. The year 1995 is included along with 1996 to illustrate the presence of outliers during periods of significant negative autocorrelation (Figure 3).

**Figure 11:**
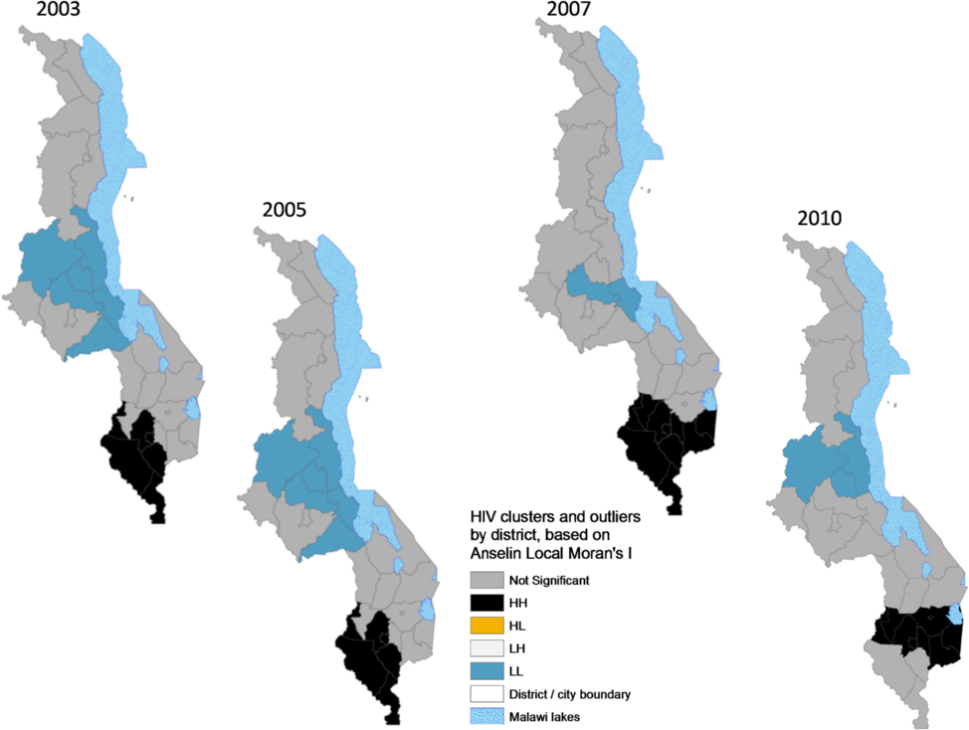
**Spatiotemporal patterns of HIV hotspots and outliers by district, 2003-2010.** Estimates of district HIV prevalence were based on the original 19 ANCs for longitudinal depth. Figure 11 shows the years 2003, 2005, 2007 and 2010.

Unpacking observed spatial patterns further through local spatial analysis revealed statistically significant clustering of districts into ‘hotspots’ and ‘coldspots’ of HIV prevalence and significant change over time. The Anselin Local Moran’s I showed core clustering of high HIV-prevalence districts next to high ones (HH) consistently located in the southern region and variously composed mainly of 11 districts (Blantyre, Blantyre City, Chikwawa, Chiradzulu, Mulanje, Mwanza, Neno, Phalombe, Thyolo, Zomba, Zomba City) (Figures 10 and 11). While relatively stable during the years that had statistically significant autocorrelation (Figure 3), the hotspot cluster, located at the southern end by 2003, had expanded to include southeastern districts of Chiradzulu, Mulanje and Phalombe by 2007 before shrinking to nine districts and shifting slightly northwards by 2010. Analysis also showed a core “coldspot” cluster of low next to low (LL) districts variously composed of six Central Region districts (Kasungu, Dowa, Ntchisi, Nkotakota, Salimaand Dedza). The coldspot cluster was largest in 2003 and 2005 but had shrunk to 2-3 districts by 2007 and 2010.Statistically significant spatial outliers (HL, LH clustering) were evident only for 1995 and 1996, years that had negative spatial autocorrelation (Figure 3). This illustrates empirically that the lack of significant global (first order) patterns of positive autocorrelation at district level for these years was because of the dominance of more localized (second order) spatial variability in HIV prevalence relative to primary order spatial patterns. Thus, Lilongwe City had exceptionally high HIV prevalence next to a low prevalence neighborhood (HL in Figure 10) in 1995 and 1996, while Zomba City had significantly low prevalence surrounded by high prevalence districts (LH). Further, these two years had the least (first order) hotspot clustering (Figures 10 and 11). These years of localized variability in HIV prevalence represent fast HIV spread (Figures 4-9), suggesting potential spatial expansionary trend of the epidemic, e.g. outward from Lilongwe City and inward into Zomba City from neighboring Blantyre City, Chiradzulu and Mulanje.

### Potential drivers of HIV prevalence for 2010 and their local spatial patterns

**Figure 12:**
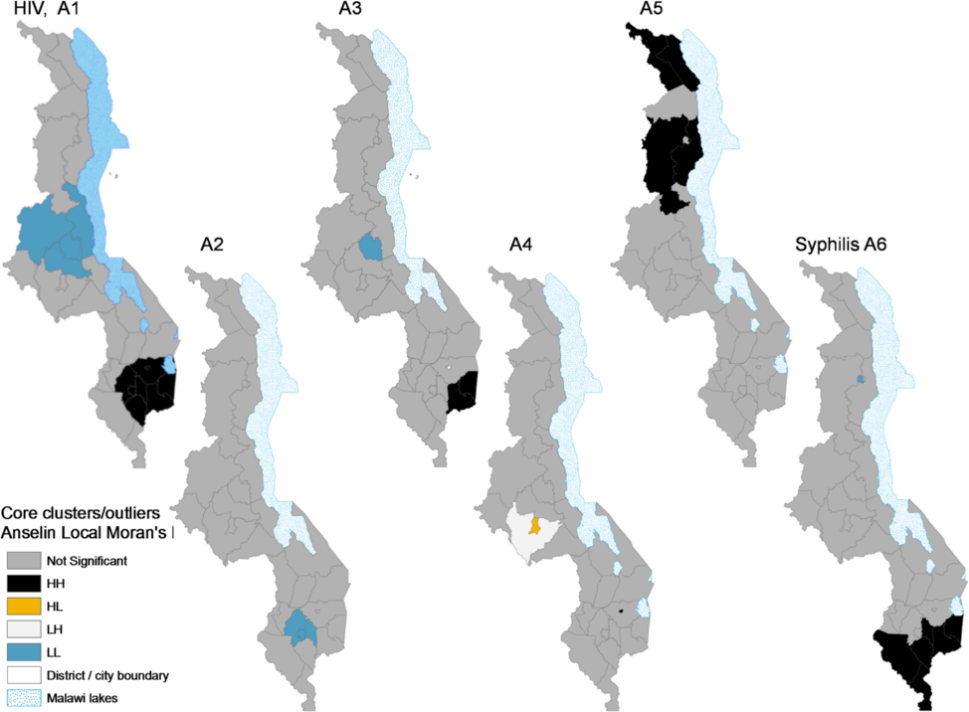
**Distribution of core spatial clusters and outliers of HIV prevalence relative to main explanatory variables.** The *core* clusters and outliers of HIV prevalence and of identified main explanatory variables are based on the Anselin Local Mora’s I. The variables displayed are: HIV, A1) 2010 HIV prevalence, A2) mean distance to main roads (km), A3) mean travel time to main public transport for the 30-44 age group, A4) percentage reporting having ever had an HIV test, A5) percentage who had attained senior primary education, and A6) 2010 syphilis prevalence (%).

**Figure 13:**
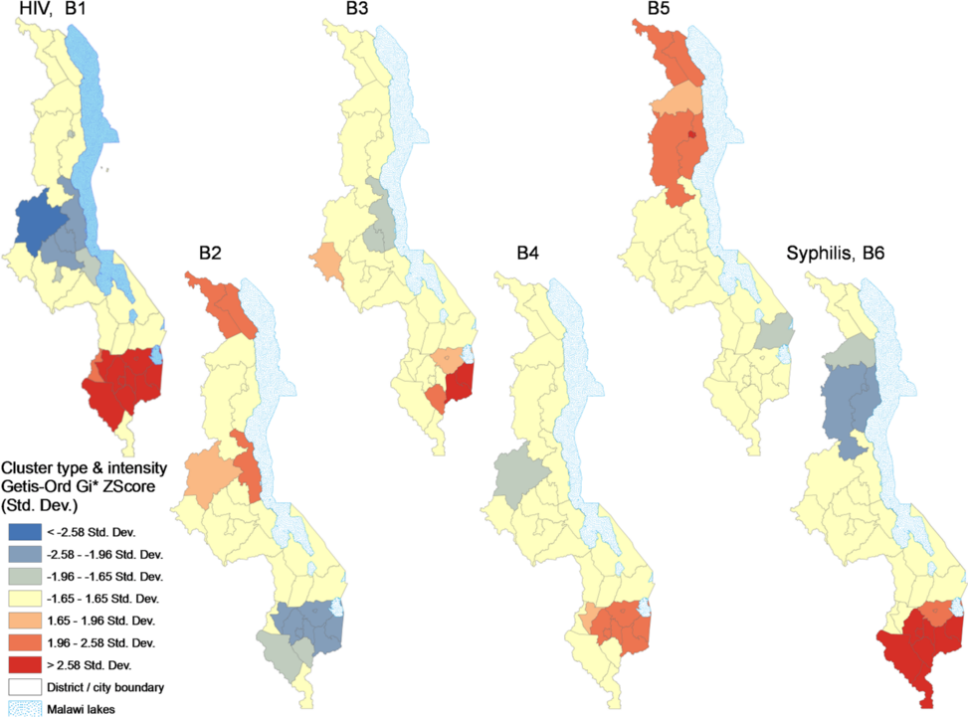
**Spatial patterns and intensity of HIV prevalence hotspots relative to patterns of main explanatory variables.** The *intensity* of hotspots/coldspots of HIV prevalence and of identified main explanatory variables are based on the Getis-OrdGi* ZScore measured in standard deviations. The variables displayed are: HIV, B1) 2010 HIV prevalence, B2) mean distance to main roads (km), B3) mean travel time to main public transport for the 30-44 age group, B4) percentage reporting having ever had an HIV test, B5) percentage who had attained senior primary education, and B6) 2010 syphilis prevalence (%).

Ordinary Least Squares (OLS) regression of estimated district-level HIV prevalence for 2010 produced four models and we chose Model 4 as the “best” model based on collinearity diagnostics and explanatory power [see Table 2]. Model 4 had four variables, each statistically significant (p = 0.024 to p = 0.000) and no significant multi-collinearity problems (tolerance 0.59-0.89 and VIF 1.12-1.69). The four variables used in all four models in Table 2 were selected through a multi-stage statistical process from an initial pool of 37 variables drawn from the literature and based on availability at district level. The model F statistic was highly significant (p = 0.000) and explained 68.8% of the variance (R^2^ = 0.64, adjusted R^2^ = 0.62).Mean travel time to nearest public transport for ages 30-44 was positively associated with HIV prevalence and had the highest influence (highest Beta value) in explaining prevalence among the four variables. The longer it took to travel to the nearest public transport, the higher the HIV prevalence at district level. Spatial analysis of this variable revealed a core HH cluster covering Mulanje and Phalombe Districts within the southern HIV hotspot, and a matching coldspot (Ntchisi district) in central Malawi. Analysis with the Getis-OrdGi* statistic added Thyolo and Zomba districts as secondary and tertiary intensity clusters, respectively. However, this variable was essentially uncorrelated (r = -0.045, p = 0.810) to the other geographic and third most influential variable in the model, mean distance to main roads. HIV prevalence decreased with mean distance from main roads. Its local spatial variation revealed a single coldspot (areas closest to main roads) capturing Blantyre City and neighboring Blantyre Rural and Chiradzulu districts. However, differentiated hotspot analysis with the Getis-OrdGi* statistic showed a secondary coldspot cluster which was closely matched with the core HIV hotspot cluster for 2010 (Figures 12 and 13).

**Table 2:** Summary of regression coefficients for possible drivers of HIV prevalence

	**Model 1**			**Model 2**			**Model 3**			**Model 4**		
**Variable description**	**Beta**	**Tolerance**	**VIF**	**Beta**	**Tolerance**	**VIF**	**Beta**	**Tolerance**	**VIF**	**Beta**	**Tolerance**	**VIF**
Attended senior primary sch. (%)										-0.287*	0.836	1.196
Percentage that had taken an HIV test ever							0.435**	0.621	1.611	0.360**	0.591	1.691
Mean time to transport, age 30_44 (min)				0.369**	0.998	1.002	0.483***	0.899	1.113	0.507**	0.892	1.121
Mean dist. to main roads (km)	0.605***	1.000	1.000	0.589***	0.998	1.002	-0.341*	0.656	1.523	-0.291*	0.641	1.560
R^2^	Model 1	0.366		Model 2	0.5		Model 3	0.62		**Model 4**	**0.688**	
R^2^ adjusted		0.344			0.467			0.467			**0.64**	
F-ratio		16.757			14.662			14.354			**14.130**	
F-ratio significance		.000			0.000			0.000			**0.000**	

N = 31 districts. Significance of the coefficients for individual variables was * for p = 0.05, ** for p = 0.001, and *** for p = 0.001 or lower. Tolerance is proportion of the variance explained by the variable alone and VIF is the Variance Inflation Factor, both collinearity diagnostic statistics. Model 4 was chosen as the “best” model.

The proportion that had ever taken an HIV test was the only behavioral, and the second most influential, explanatory factor for HIV prevalence, and was (counterintuitively) positively associated with HIV prevalence (Table 2). Only Zomba city emerged as a core hotspot for this variable and Lilongwe City as a spatial outlier (Figure 12). The percentage of those who had taken at least an HIV test was highest in Lilongwe city (81.9% of adults, hence an HL outlier) in relation to the exceptionally low Lilongwe Rural (52.9%, hence an LH outlier). However, analysis with the Getis-OrdGi* statistic revealed a secondary intensity hotspot cluster (GiZScore = 1.96-2.58 SDs) perfectly spatially matching the eight core HIV hotspots for 2010.

Education, in particular the percentage of the population that had attended senior primary school (grades 6-8), was significantly negatively correlated with HIV prevalence, although it had the lowest Beta value among the four “best” model variables. This suggests that the more pregnant women who have a little education (senior primary) the less likely they are to have HIV/AIDS. Cluster analysis for this variable shows only three northern districts (Chitipa, Karonga and Mzimba) standing out as hotspots. Analysis with Getis-OrdGi* statistic adds two secondary hotspot districts (Nkhata Bay and Mzuzu City) and one coldspot district. None of the HIV hotspot districts were in education “hotspots.”

## Discussion

### HIV clustering and spatiotemporal patterns of HIV prevalence in Malawi

This study has provided a visually powerful and empirically derived, multi-scalar analysis of spatiotemporal variation in HIV prevalence among pregnant women attending ANCs in Malawi from 1994 to 2010. It goes beyond current broad characterizations at national, regional and urban/rural variation in HIV prevalence (Figure 2) to local variation at district and lower (continuous) levels (Figures 4, 5, 6, 7, 8, 9, 10, 11, 12, and 13). GIS tools allowed the generation of HIV prevalence estimates at scales where HIV data are not traditionally collected (district and continuous) from point HIV sentinel data at 19 ANCs for spatiotemporal pattern analysis and from 54 ANCs for use in multivariate analysis of possible drivers and their spatial variation. Observed widespread spatial variation in HIV prevalence reveal the HIV epidemic as an aggregation of several spatially defined sub-epidemics - national, regional, urban, rural, and local (clusters). Findings broadly call into question the use/effectiveness of one-size-fits-all interventions and policies under such circumstances.

The most prominent temporal trend was the general decline in HIV prevalence after rapid spread/intensification up to 1999. For Malawi, the trend positively reflects on the concerted planning and considerable human and financial resources (most from donor aid) applied to prevention and treatment efforts by government and other agencies over the past decade [17]. However, the impressive rates of national decline in HIV prevalence among ANC-attending pregnant women are 1) slowing down from 1.1% annually betweeen1999 and 2010, to 0.91% and 0.88% annually for the periods 2003-2010 and 2005-2010, respectively (Figures 4-11) tempered by a modest decline in the general population (from 12.0% in 2004 to 10.6% in 2010, 0.2% annually) based on MDHS data [14] - a cautionary tale against complacency.

Another significant spatiotemporal trend was a slow but emergent spatial evening in HIV prevalence as the epidemic stabilized and then declined after an initial period of localized spatial heterogeneity and explosive spatial spread and intensification to a peak in 1999, as illustrated empirically in temporal patterns of global Moran’s I (Figure 3) and in various ways in Figures 4-11. First, there was a general narrowing in the prevalence gap among the regional sub-epidemics (more so for the north and center, and between the urban/semi-urban and rural epidemics. Second, the negative autocorrelation evident in 1995 and 1996 indicated localized heterogeneity with high next to low prevalence pockets confirmed as spatial outliers in clustering patterns (Figures 10 and 11), which are generally associated with expansionary spatial processes and rapid spread [54]. Subsequent increases in the level and significance of positive spatial autocorrelation (Figure 3) further indicate *relative* spatial evening of the epidemic with districts of similar HIV prevalence more clustered together. The (low) decrease in total statistically significant clustering (12 core hotspot and coldspot cluster districts 2001 to 2010 relative to 14 in 1994 and 1999) also indicates spatial evening as districts that stand out from the rest decrease. The fact that the spatial and population center of gravity was firmly located in rural areas where the majority (85%) of Malawians lived [49] and the HIV epidemic was more stable and lower in intensity (at least among pregnant women), may have provided the inertia that kept the national epidemic in relative check. The rural epidemic’s declining trend may thus be a key turning point nationally.

Identification of a hotspot cluster of 5-11 districts making up the HIV epicenter consistently located in the south was the most significant outcome of cluster analysis. Potential explanations include the long history of urbanization in the south forming a hub for HIV transmission. Cluster analysis revealed a hierarchy among the four biggest cities in the country in HIV prevalence relative to surrounding districts. Blantyre, the first city in Malawi and the commercial capital, anchored the HIV hotspot/epicenter every year of analysis, followed by Zomba (twice in a hotspot cluster), then Lilongwe (twice an HL outlier but part of a primary or secondary coldspot in most of the study years), and then Mzuzu city [Figures 10 and 11]. The Southern Region was also the most densely populated [49], and had the highest levels of rural poverty [50] and prevalence of syphilis [17]. The 2010 MDHS also showed that women and men in the Southern Region had the highest percentage of those who had two or more sexual partners in the previous 12 months and mean number of sexual partners per lifetime, while men had the lowest percentage reporting using a condom during last sexual intercourse (22.9%) [14]. Elevated labor migrancy, particularly returning Malawian mine workers from South Africa late1980s/early 1990s, may partly explain emergence of an HIV sub-epicenter from 1994 to 1999 (Figures 6-9) among the northern districts of Nkhata Bay and Rumphi, and Mzuzu city [32,55].

### Potential drivers of HIV prevalence for 2010 and their local spatial patterns

Geographic variables, particularly mean travel time to nearest road, had the most explanatory influence on HIV prevalence among the variables in the “best” regression model (Table 2), but the two geographic variables in the model were virtually uncorrelated and they appeared to capture subtly different aspects of “access”. The spatial variation of these two variables in relation to HIV prevalence (and hotspots) also differed. Main roads generally link places to main towns and cities and this variable mainly captured the urban/rural divide. Increasing distance from main roads reflected increasing distance from urban areas and the factors that elevated HIV prevalence there, while increasingly capturing more isolated and sparsely populated areas with limited mobility and sexual mixing and access to health services and conditions that are generally associated with lower risks of HIV infection. This explains the negative relationship with HIV prevalence. Cluster analysis of distance from main roads revealed only one statistically significant core cluster, a coldspot (districts exceptionally close to main roads and urban areas, hence high HIV risk areas) cluster of Blantyre city and neighboring Blantyre Rural and Chiradzulu districts. This suggests that distance to cities (or urban influences) is a major explaining factor for high HIV rates in this subset of the core HIV hotspot districts, although hotspot analysis with the Getis-OrdGi* statistic expanded the primary coldspot cluster to 8 districts which virtually matched the larger core HIV hotspot.

In contrast, core and secondary hotspots for mean travel time to nearest transport (30-44 age group), which varied positively with HIV prevalence, matched three rural HIV hotspot districts of Mulanje, Phalombe and Thyolo spatially. These districts (including Zomba Rural as a tertiary HH cluster) have hilly terrain, which is a physical obstacle to travel and access to health services. Further, high concentration of commercial farms (mainly tea) forced many people into smaller remaining areas, making for some of the highest population densities in relation to available arable land. The dense populations, including the predominantly male commercial farm workers (a high HIV risk group in Malawi [56]), facilitated increased sexual mixing and risky sexual encounters, and HIV spread. These areas need increased access to HIV information and other services, including through mobile services.

Our findings are generally consistent with recent studies elsewhere in Africa. Tanser et al. [1] also found an inverse significant relationship between HIV prevalence and distance to the main road in Kwazulu-Natal, South Africa. Travel time to nearest public transport captures a similar relationship in the inverse direction (longer/farther from transport, lower HIV rates). Studies reveal a mixed role of education, suggesting that it is mediated by complex factors. The 2004 and 2010 MDHS studies show the level of education generally positively associated with HIV prevalence [14]. However, Moise and Kalipeni found a negative relationship for literacy rates above 20% in Zambia based on a spatial lag model. Given our more specific definition of education, and controlling for HIV testing, distance to main roads and time to main transport, the negative relation with the percentage of those who had attained this modest (senior primary education) suggests some level of education is good for AIDS prevention.

Further, we found that the percentage of people in the larger population (men and women) who had taken at least one HIV test ever was positively associated with HIV prevalence. The secondary hotspot clusters for HIV testing revealed by the Getis-OrdGi* statistic captured seven districts that closely matched the core HIV prevalence hotspot for the women’s ANC sample for 2010 (Figure 13, B4). This finding that levels of HIV testing were highest in districts that already had high HIV prevalence is plausible because the sense of risk and value of HIV testing would likely be heightened in such areas. Indeed, the 2011 Malawi Welfare Monitoring Survey found that the main reason given by 44% of non-tested respondents for not testing was that they did not feel at risk or in need of an HIV test [51]. During Malawi’s 2010/11 fiscal year, 1.773 million people (28% of the sexually active population) were tested for HIV in Malawi [12]. However, HIV testing was higher among urban residents who are generally wealthier and more educated, have better access to testing facilities, but also a higher risk of being infected than rural residents [3,6,14]. This relationship explains the emergence of Lilongwe City as a core HL outlier and Zomba City as a core hotspot (Figure 12, A4). However, Blantyre City does not appear as an outlier probably because the core HIV cluster in the south is more extensive and includes Blantyre and several (rural) districts (Figure 12, HIV, A1). Further nuanced analysis may be needed on HIV testing behavior and its impact on HIV prevention.

Although the four variables included in the “best” model explained a relatively high proportion of the variance in HIV prevalence (68.8%, see Table 2), closer examination suggests that these variables were essentially proxies for underlying factors related to geographic/physical and socio-economic access or exposure to health services and other amenities, HIV prevention knowledge, personal resources, sexual networks and risky sex and other factors that influence HIV-related behavior and risk, mainly mediated by urban/rural residence or proximity and poverty/wealth and education. Further, some variables not included in the final model were interesting. For instance, the core and secondary clustering patterns (hotspots) for 2010 syphilis prevalence, highly significant in binary correlations (Table 1), were a near-perfect match with HIV hotspot districts (Figures 12 and 13), suggesting that syphilis treatment/management also needs special attention. Therefore, more detailed analysis needs to be done, focusing on local HIV variation at finer spatial scales and drivers of HIV *incidence* to better inform policy, rather than on prevalence as was done in this study. Nevertheless, HIV incidence is often closely associated with prevalence, and as in Tanser et al. [1], we also assumed that time lags between cause and effect would not significantly model outcomes given our emphasis on preliminary associations.

### Limitations of the study

While this study contributes significantly to advancing spatiotemporal analysis of HIV/AIDS in Malawi, Africa, and the developing world generally, it has limitations. The longitudinal depth of HIV sentinel data and caution with the interpolation process including consistency across years allowed adequate assessment of spatiotemporal patterns/trends among pregnant women, but the small number (19) of sentinel ANCs, used in the spatiotemporal analysis means that the interpolated products and especially the derived district estimates are and should be treated as indicative, although the HIV data for the regression analysis were from a larger sample (54 points). Local spatial-statistical pattern (hotspot) analysis also came with uncertainties, including the choice of method or search distance to define a neighborhood. In this study, we empirically determined it as the distance at which global Moran’s I was highest for most years, 80 km. Further research is needed to determine the spatial limit of the spatial dependence of HIV on neighboring values at different spatial scales to better guide interventions. Finally, although our choice of an OLS regression model in the presence of spatial dependence breached the assumption of independence of measurements, OLS was sufficient for the study purpose of identifying indicative explanatory factors for observed spatial variation (as opposed to producing predictive models). Moreover, a corrective spatial lag model of HIV prevalence using standardized variables in the “best” OLS model based on spatial diagnostics was performed within the GEODA spatial statistical software [53,57]. Despite improvements in the explanatory power of the model and significance of coefficients for all but one of the four independent variables (the p-value for distance to roads fell slightly from 0.043 to 0.06), the spatial lag model did not change the directions of the variable relationships nor their relative importance.^f^ In the interest of space, we left out the spatial lag model findings.

## Conclusion

This study has 1) shown spatiotemporal trends in HIV prevalence at multiple scales (national, regional, district and sub-district/continuous) in Malawi from 1994 to 2010 using spatial analysis of data from pregnant women attending HIV sentinel surveillance centers and GIS tools and 2) identified five socio-demographic, behavioral, socio-biological and geographic variables found to be significantly associated with HIV prevalence in multiple ordinary least squares (OLS) regression analysis and mapped their spatial variation at district level in relation to the spatial distribution of HIV hotspots, coldspots and spatial outliers. A varying core hotspot of 6-11 districts was found in the south and a coldspot of 1-6 districts in the center, but the epidemic was slowly leveling out spatially in terms of prevalence. Findings illustrated the importance of spatially explicit geographic analysis to enhance understanding of the spatial and temporal variation and nature of the HIV pandemic along with configurations of factors that shape it in particular locations, with potential to enhance spatial targeting of HIV interventions and policies.

The results indicate that for Malawi there were several geographically differentiated HIV/AIDS epidemics rather than a single one. Further, the results of our analysis offer the most spatially explicit longitudinal analysis of HIV prevalence in the country that we are aware of. The study joins a small but growing number of studies with similar spatial specificity including mapping the spatial variation of proximate and underlying factors that influence HIV prevalence. Despite acknowledged shortcomings associated with limited sample size, this study demonstrates the broader importance of using explicit spatial analysis to understand the geographic nature of the epidemic, examine spatiotemporal trends, and use this knowledge for more effective spatial targeting to combat the HIV/AIDS pandemic.

## Endnotes

^a^The presence of spatial dependence implies that HIV prevalence values in one district are similar to and dependent on values in neighboring districts. It is also called spatial autocorrelation.

^b^However, analysis of spatial disease clustering is more common with other diseases in developed regions of the world [1], with some on HIV/AIDS (e.g., [19]).

^c^Very localized analysis has included issues of access to HIV anti-retroviral treatment in part of Karonga district, northern Malawi [22].

^d^Target sample sizes are 300, 500, and 800 women at rural, semi-urban, and urban ANCs, respectively.

^e^Concerns involve sample representativeness –use of purposive sampling favoring urban/high risk areas over rural ones, paucity of ANCs, and narrow population of pregnant women attending ANCs relative to the larger population. Limited access and historical depth reduce utility of emerging population-based HIV survey data, e.g. the MDHS.

^f^The R square increased from 0.688 to (pseudo) 0. 837, the Log likelihood increased from -70.757 to -61.932, while the Akaike info criterion and Schwartz criterion decreased from 151.515 to 135.863 and 158.685 to 144.467, respectively. These changes are indicative that the spatial lag model is better than the OLS model, and confirm that space matters as a factor in HIV prevalence among pregnant women attending antenatal clinics in Malawi [57].

## Abbreviations

ANC: Antenatal clinic; CI: Confidence interval; DHS: Demographic and health survey; DRC: The Democratic Republic of the Congo; GIS: Geographic information systems; GoM: Government of Malawi; HH: High-high hotspot (clustering of districts with outstandingly high HIV or other values); HF: Health facility; HL: Hi-Low hotspot (an outlier high-value district surrounded by low value ones); HTC: HIV testing and counseling; IDW: Inverse distance weighted; IHS: Integrated health survey; LISA: Local measures of spatial autocorrelation; LH: Low-High outlier (a coldspot district surrounded by high value ones); LL: Low-Low coldspot (clustering of districts with very low values); MDHS: Malawi demographic and health survey; MK: Malawi Kwacha (Malawi’s currency); MoH: Ministry of health; NAC: National AIDS Commission; NSO: Malawi National Statistical Office; OLS: Ordinary least squares; RMSE: Root mean square error; SD: Standard deviations; STI: Sexually transmitted infection; UNAIDS: The Joint United Nations Program on HIV/AIDS; WHO: The World Health Organization; WMS: Welfare monitoring survey.

## Competing interests

Authors have no competing interests to report in relation to this article.

## Authors’ contributions

LCZ conceived the study, conducted the GIS and statistical analysis and wrote the first draft. EK participated in the conceptualization of the study, contributed to the literature review and revisions of the draft manuscript. EMJ participated in the design of the study and helped to draft and review the manuscript. All authors approved the final manuscript.

## Pre-publication history

The pre-publication history for this paper can be accessed here:

http://www.biomedcentral.com/1471-2334/14/285/prepub
